# Hypothesis: A Challenge of Overexpression *Zfp521* in Neural Tendency of Derived Dental Pulp Stem Cells

**DOI:** 10.22074/cellj.2019.5600

**Published:** 2018-11-18

**Authors:** Fatemeh Behrouznezhad, Fatemeh Ejeian, Modjtaba Emadi-Baygi, Parvaneh Nikpour, Mohammad Hossein Nasr-Esfahani

**Affiliations:** 1 Department of Genetics, Faculty of Basic Sciences, Shahrekord University, Shahrekord, Iran; 2 Department of Cellular Biotechnology, Cell Science Research Center, Royan Institute for Biotechnology, ACECR, Isfahan, Iran; 3 Department of Genetics and Molecular Biology, Faculty of Medicine, Isfahan University of Medical Sciences, Isfahan, Iran

**Keywords:** Mesenchymal Stem Cell, Neurodegenerative Diseases, Neuronal Differentiation, Zinc Finger Protein 521

## Abstract

Neurodegenerative diseases have now become a major challenge, especially in aged societies. Most of the traditional strategies used for treatment of these diseases are untargeted and have little efficiency. Developments in stem cell investigations have given much attention to cell therapy as an alternative concept in the regeneration of neural tissues. Dental pulp stem cells (DPSCs) can be readily obtained by noninvasive procedures and have been shown to possess properties similar to well-known mesenchymal stem cells. Furthermore, based on their neural crest origin, DPSCs are considered to have a good potential to differentiate into neural cells. *Zfp521* is a transcription factor that regulates expression of many genes, including ones involved in the neural differentiation process. Therefor based on neural crest origin of the cell and high expression of neural progenitor markers, we speculate that sole overexpression of Zfp521 protein can facilitate differentiation of dental stem cells to neural cells and researchers may find these cells suitable for therapeutic treatment of neurodegenerative diseases.

Neurodegenerative diseases have the profound impacts on modern human life, which common treatment in the medical field often fail. This event is mainly attributed to the limited ability of the adult central nervous system (CNS) for regeneration of neurons and glial cells (1, 2). Therefore, this dearth has lead researchers in the nascent field of regenerative medicine to assess the ability of different types of stem cells, including adult stem cells (ASCs) and embryonic stem cells (ESCs) to differentiate into neurons (2-4). Despite the high ability of ESCs to self-renewal and also to differentiate into various types of cells, the risk of teratogenicity, rejection, and ethical issues narrow their medical application. In contrast to ESCs, these risks are less associated with ASCs that show lower degree of plasticity. In this regard, different approaches are used to improve the transdifferention potential of ASCs to applying in regenerative medicine (2, 4).

Dental stem cells (DSCs) originated from the cranial neural crest and reside in different parts of the oral cavity. These cells are easily obtained by relatively noninvasive methods and can differentiate into neurons, chondrocytes, cardiomyocytes and osteoblasts cells (5). During the last decade, many studies have revealed that these cells also express key neurotropic factors including neurotrophin 3 (NT3), brain-derived neurotrophic factor (BDNF) and neurotrophin 4 (NT4) as well as some of the main neural markers such as Nestin, Sox2 and Glial Fibrillary Acidic Protein (GFAP) (6-11). Previously, an exciting experiment revealed a relatively high similarity in DNA methylation pattern in dental pulp stem cells (DPSCs) and some neural stem cell lines, which confirmed their neural regeneration plasticity and their common origin. So, it is believed that these cells have an innate tendency to differentiate into neurons, which can be augmented by exogenous transcription factors (1, 12). 

Zinc finger protein 521 (Zfp521, also known ZNF521 in human) is a highly conserved nuclear factor that contains 30 Kruppel-like zinc finger motifs and different co-regulatory domains. As a result, Zfp521 is capable of interacting with many transcriptional cofactors (13, 14) in diverse developmental processes and is involved with nucleosome remodeling in various tissues and organs (15-17).

Furthermore, it has been proven that Zfp521 shares a common 12 amino acid motif with many transcriptional repressors, like nucleosome remodeling and deacetylase (NuRD). A significant amount of Zfp521 protein in osteo/chondro progenitor cells recruits NuRD and some other histone deacetylases (HDCAs) that consequently attenuates *RUNX2*, as a specification gene (18-20).

**Fig.1:**
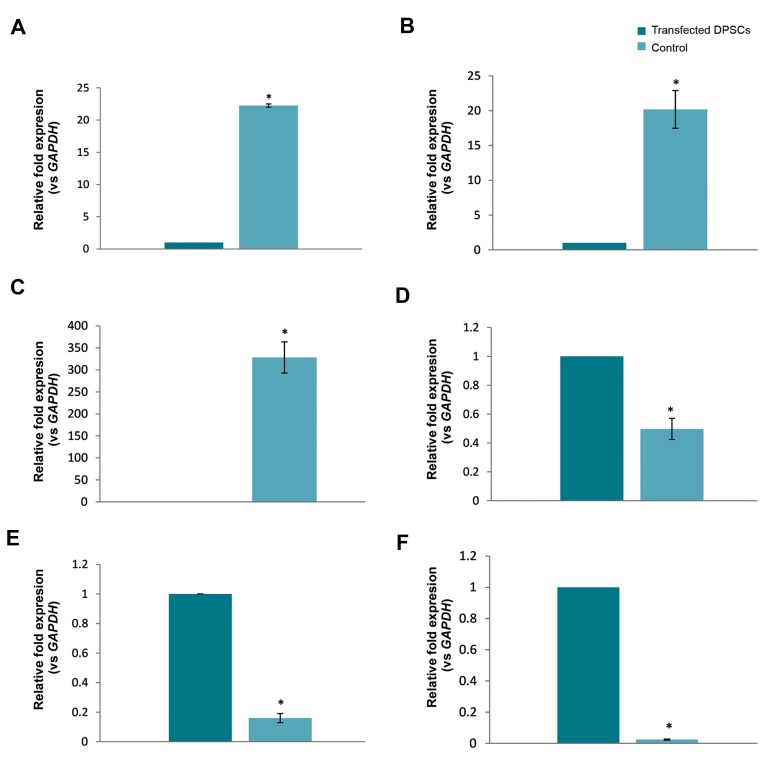
Expression of some genes related to Zfp521. **A**. The higher level of Zfp521 experrsion detected in trasfected cells in comparison with untraffected dental pulp stem cells (DPSCs), **B**. Zfp521 overexpression resulted to the significantly up-regulation of SOX3, C. PAX6 as neural progenitor markers, while induced down-regulation in D. CDK1 expression as a key player in cell cycle progression and non-neural determination genes such as E. PPAR-γ, and F. BMP2 that reflected adipogenesis and osteogenesis respectively (P≤0.05).

Several studies demonstrated that Zfp521 is highly expressed in the cerebellum, striatonigral neurons and neural stem cells. In this regard, Kamiya et al. (4) showed a pronounced expression of *Zfp521* in the neuroectoderm of the rostral neural tube during neurulation, which play a key role in the conversion of ES cells into the neural progenitors. They also found that during neural differentiation Zfp521 acts in cooperation with the P300 activator via its N-terminal zinc-finger motifs and induces expression of many early neural genes, such as *SOX1, SOX3,* and *PAX6*. In this regard, *Shahbazi* et al. (21). verified that *Zfp521* has the potential to directly convert human fibroblasts into neural progenitor cells. These cells are capable of surviving, migrating, and achieving neural phenotypes upon transplantation into the neonatal mouse and adult rat brains without tumor formation. Generally, there is considerable evidence that Zfp521 acts in association with its close paralog Zfp423, at least in part, for various explained functions (22, 23).

Recently, more attention has been paid to dental stem cells as a promising source of cells for the regeneration of various tissues due to availability, ectomesenchymal origin, and a relatively high level of neural progenitor markers. Despite many reports on the effective neural induction in DSCs, little success were achieved to produce clinically applicable neurons (24, 25).

Considering all the aforementioned promising features of the DSCs, to pave the way for the application of DSCs to challenging neurodegenerative disorders through neural regeneration in future, we will propose that temporal overexpression of Zfp521 may efficiently leads DSCs to differentiate into functional neurons under specific culture conditions.

Previous studies have been revealed that epigenetic modifications have high impacts on the regulation of gene expression during neurogenesis (26). We believe that Zfp521 can mediate remodeling of nucleosome through recruitment of P300 in neural progenitor cells, which in turn promotes activation of neuron specification genes, like *SOX3* (4). The intrinsic histone acetyltransferase (HAT) activity of P300 co-activator on neural genes (27) and co-repression of histone deacetylase (NuRD) complex on some sets of non-neural determination genes, such as *RUNX2* or *SOX9*, via interaction with Zfp521, are suggested as the main mechanism involved in the neural induction effect of Zfp521.

Furthermore, Zfp521 can promote cell cycle transition from precursor to post-mitotic state via down regulating cyclin dependent kinase 1 (*CDK1*) (28). Some recent studies provided evidences for the sequential switch of chromodomain-helicase-DNA-bindings (CHDs) in NuRD complex during neural progenitor proliferation and cortical layer specification, which can be further considered as a promoter of *Zfp521* action (29).

Based on this speculation, we expect to observe higher efficacy of trans-differentiation of DPSCs after single transduction of *Zfp521* in comparison to previously reported fibroblast induction by Shahbazi et al. (21). In this regard, to provide an evaluation for this hypothesis we assessed the impact of *Zfp521* on some important genes such as *SOX3, PAX6, CDK1, PPAR-γ* and *BMP2,* which supported the neural induction potential of *Zfp521* in mesenchymal stem cells. 

Gene expression analysis was performed by real time polymerase chain reaction (PCR) after transduction of characterized DPSCs with a doxycycline inducible lentiviral vector and induction of *Zfp521* overexpression for 2 days. We found a significant increase in *Zfp521* expression in comparison to untransfected cells, which was accompanied by significantly acceleration in expression level of two main neural markers, *SOX3* and *PAX6* (Fig .1). In contrast, it seems that the overexpression of *Zfp521* not only resulted to the considerable reduction in CDK1 but also inhibited the expression of *PPAR-γ* and *BMP2* which related to adipogenesis and osteogenesis, respectively. These data provide primary evidence in support of neural inductive potential of *Zfp521*, especially for dental stem cells.

Due to remarkable potency and their neural crest origin, DPSCs are considered to have a potential to differentiate into neural cells. Although numerous studies in the last decade focused on the neural differentiation of DPSCs, the extension to functional nerve cells remains a challenge. In conclusion, we speculate that the temporal overexpression of *Zfp521* in dental pulp stem cells may prime cells for neural differentiation through chromatin modification that can lead to the expression of neural specification genes. Suggested mechanism of this effect is schematically presented in Figure 2. This proposed hypothesis should be evaluated in the neural differentiation progress to assess the neurogenesis efficiency in *Zfp521* overexpressed in these cells. Further studies of involved cellular mechanisms and proteins interaction with *Zfp521* are also valuable.

**Fig.2:**
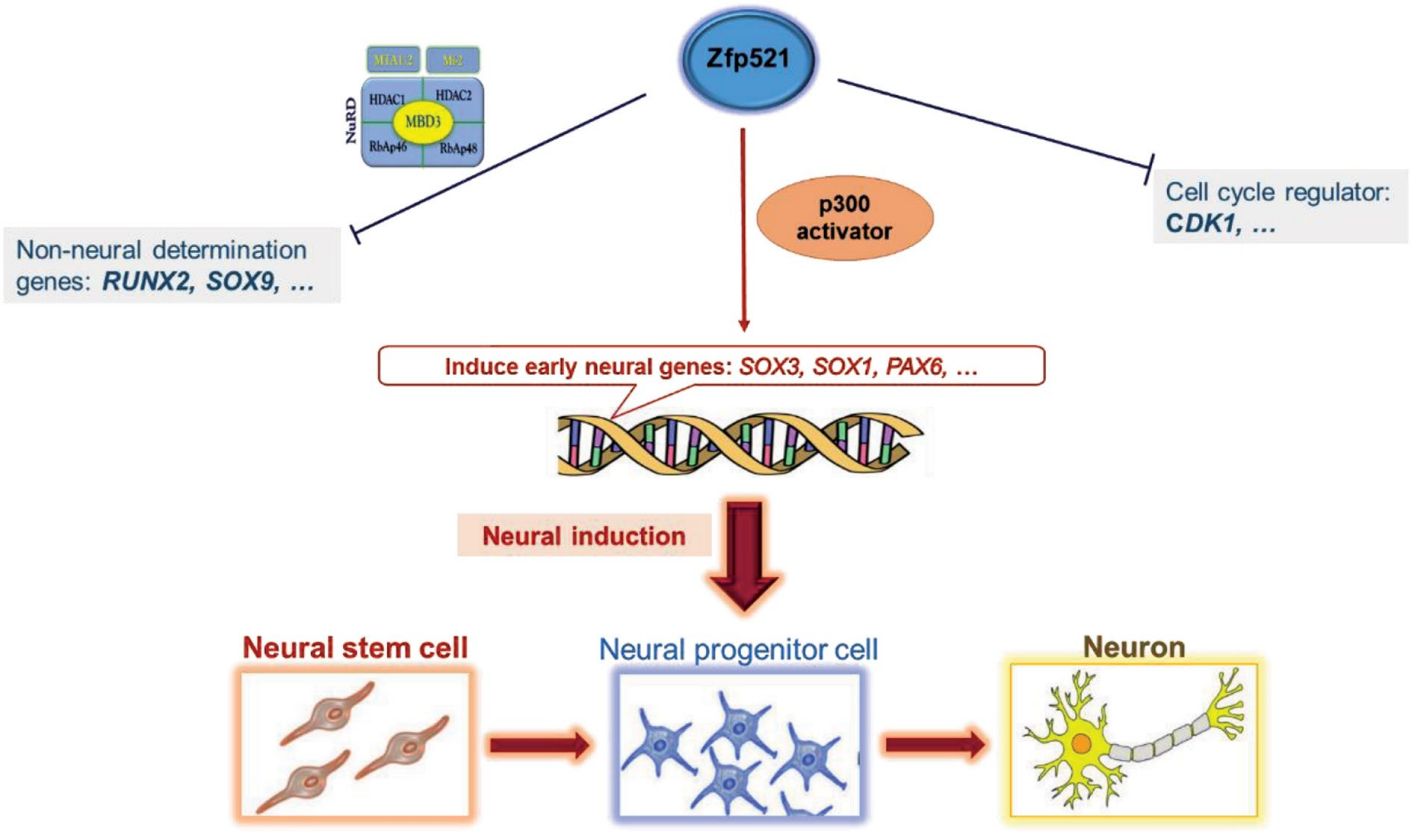
Proposed role of Zfp521 in induction of neural differentiation through mesenchymal stem cells.
